# Supplementary material for: PRMT5 Mediated HIF1α Signaling and Ras-Related Nuclear Protein as Promising Biomarker in Hepatocellular Carcinoma
Source: Biology (Basel). 2024 Mar 27;13(4):216. doi: 10.3390/biology13040216 (PMC11048327; doi:10.3390/biology13040216)
Supplement: Supplementary file 1 [file biology-13-00216-s001.zip › biology-2907798-supplementary.pdf]

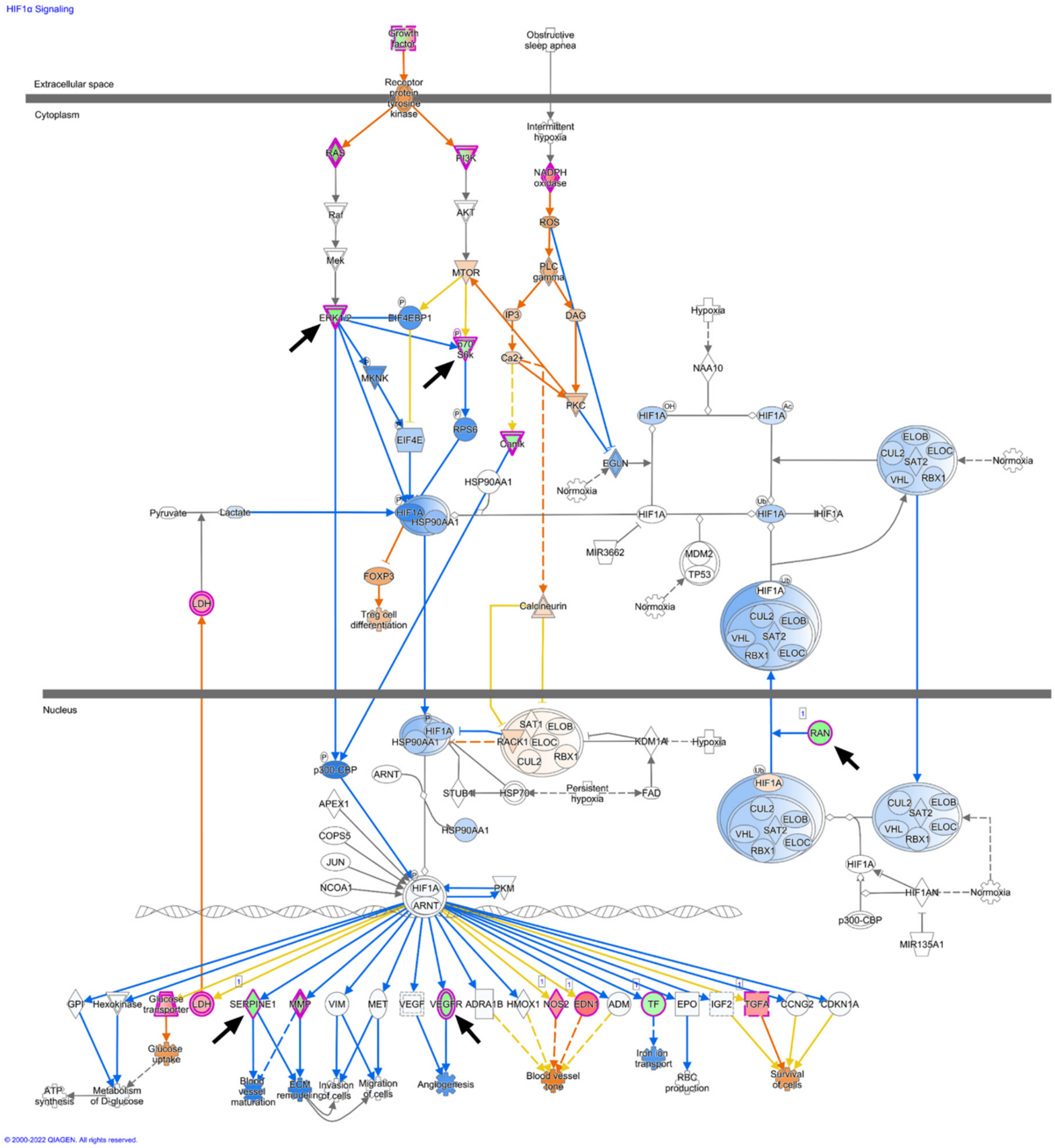

Supplementary Fig. 1: PRMT5 expression positively correlates with HIF1α signaling pathway. IPA visual diagram of the HIF1α pathway illustrates the location and predicted changes in the pathway molecules in the HHJ-7 cell line upon PRMT5 knockdown. Differentially expressed genes are marked purple. Green color refers to decrease expression while red color refers to increased expression. Molecular activity variations are highlighted orange for activation and blue for inhibition.

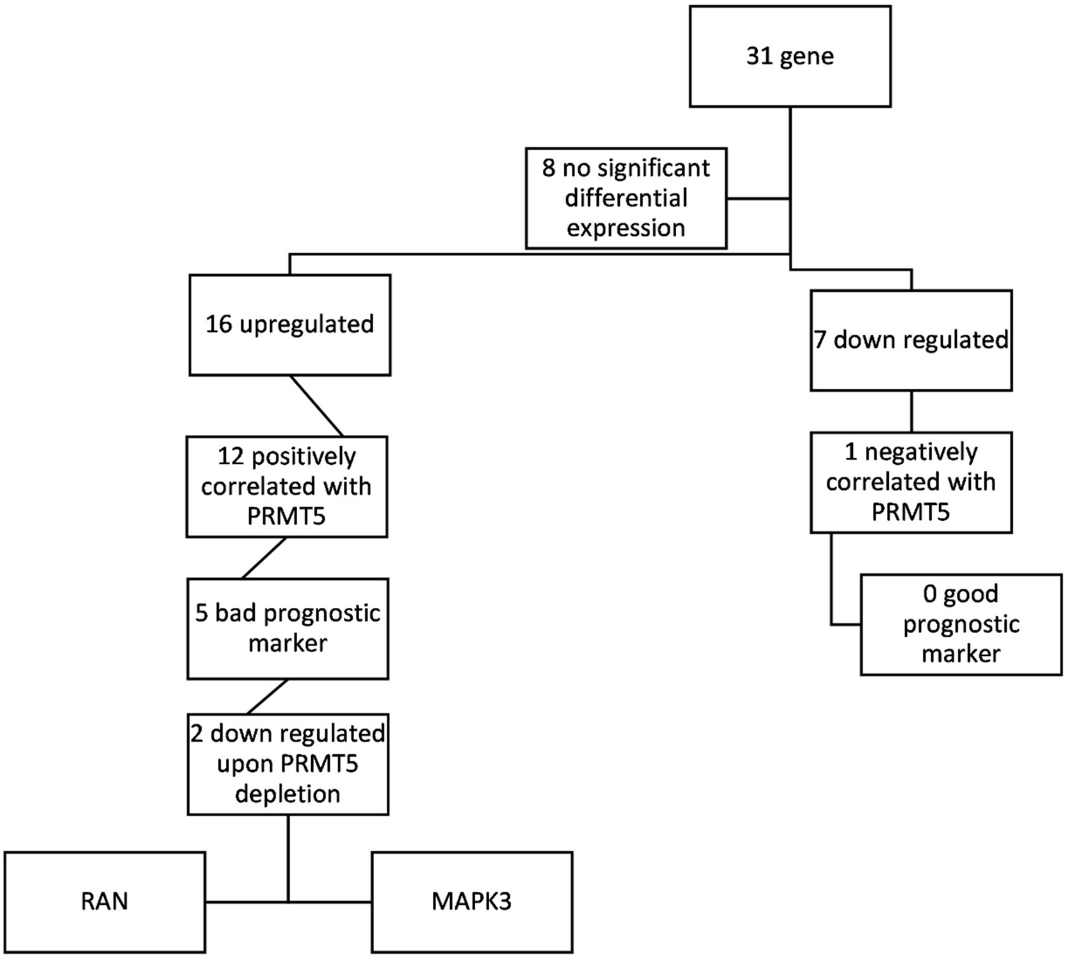

Supplementary Fig. 2: filtration criteria of HIF1α signaling pathway genes controlled by PRMT5 expression to identify potential biomarkers.

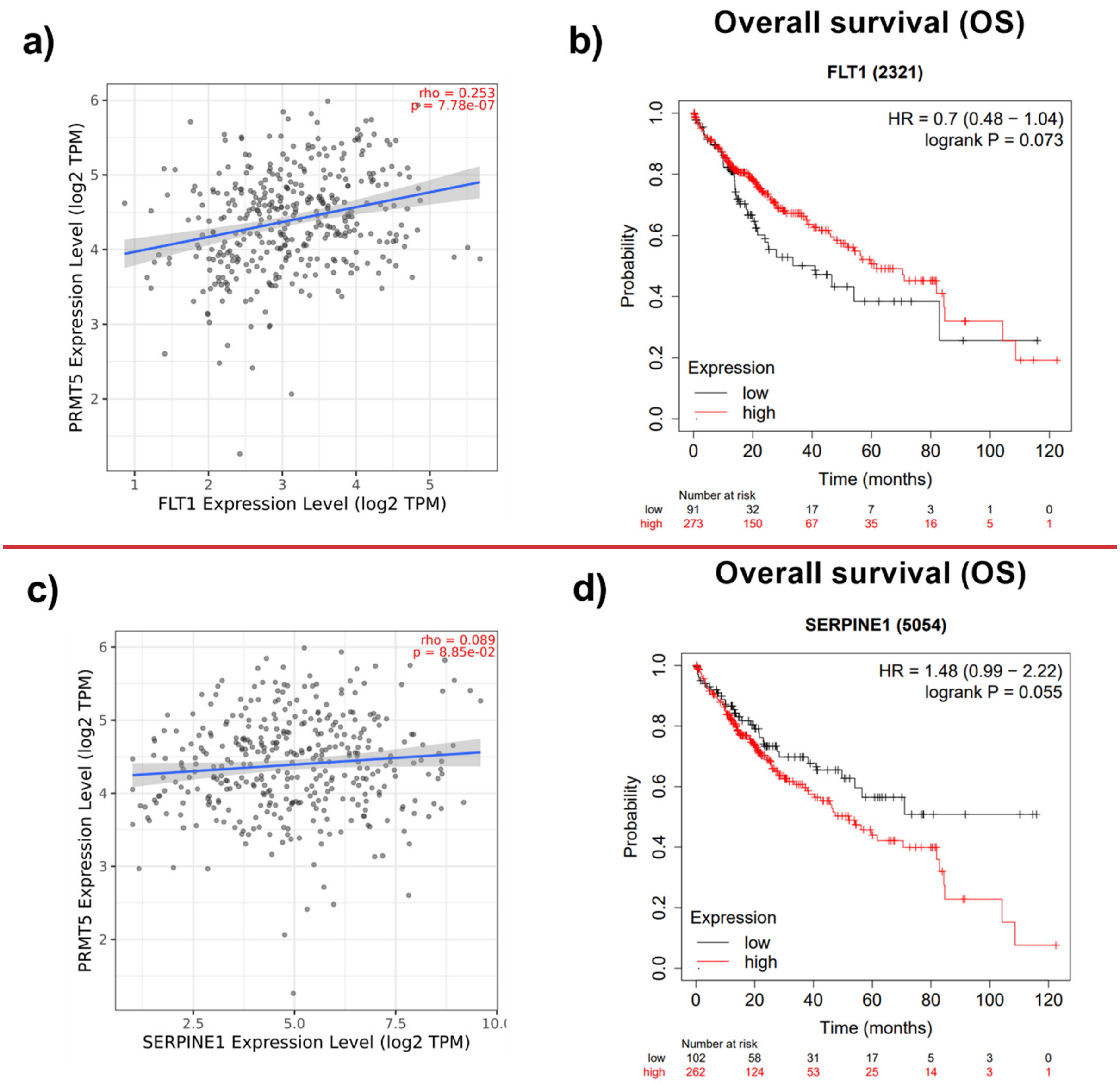
a)
rho = 0.253
p = 7.78e-07
PRMT5 Expression Level (log2 TPM)
FLT1 Expression Level (log2 TPM)
b)
Overall survival (OS)
FLT1 (2321)
HR = 0.7 (0.48 − 1.04)
logrank P = 0.073
Probability
Expression
low
high
Time (months)
Number at risk
low 91 32 17 7 3 1 0
high 273 150 67 35 16 5 1
c)
rho = 0.089
p = 8.85e-02
PRMT5 Expression Level (log2 TPM)
SERPINE1 Expression Level (log2 TPM)
d)
Overall survival (OS)
SERPINE1 (5054)
HR = 1.48 (0.99 − 2.22)
logrank P = 0.055
Probability
Expression
low
high
Time (months)
Number at risk
low 102 58 31 17 5 3 0
high 262 124 53 25 14 3 1

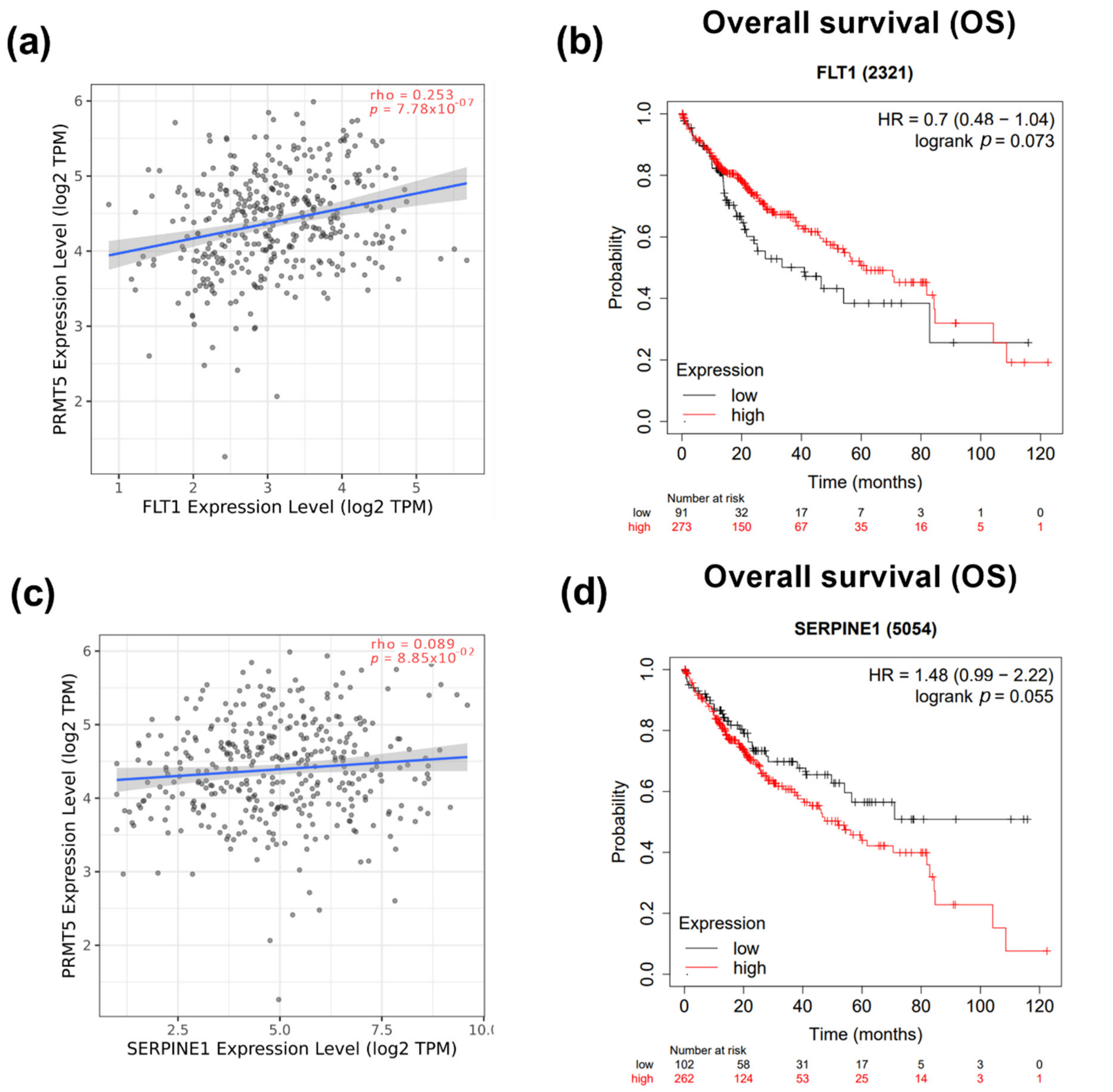

Supplementary Fig. 3: Correlation of angiogenesis related genes expression vs. PRMT5 expression and patient survival in HCC. **(a)** Spearman's rank correlation coefficient of PRMT5 versus FLT1 expression **(b)** FLT1 expression versus overall patient survival (OS) **(c)** Spearman's rank correlation coefficient of PRMT5 versus SERPINE1 expression **(d)** SERPINE1 expression versus overall patient survival (OS).

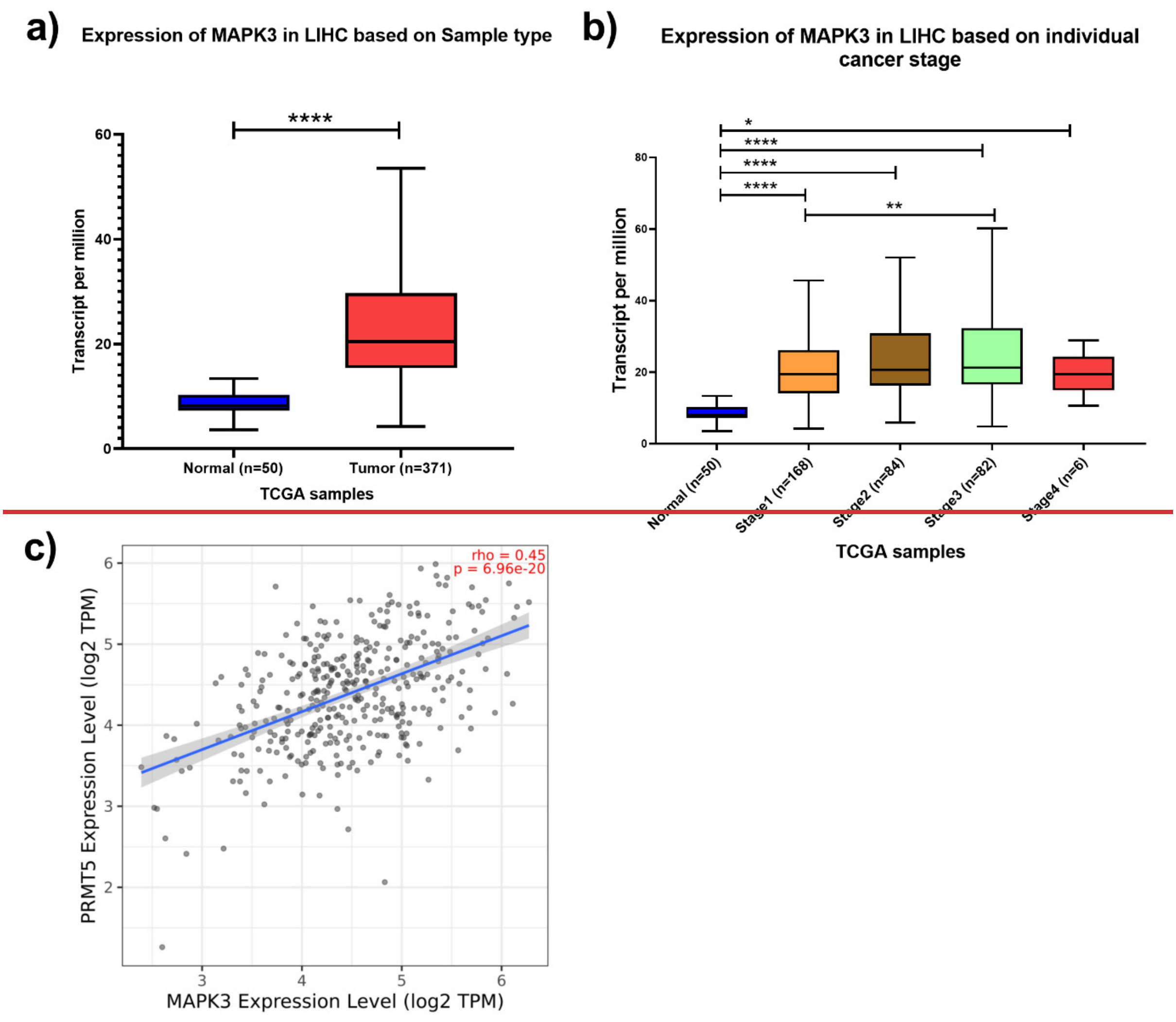
a)
Expression of MAPK3 in LIHC based on Sample type
****
Transcript per million
60
40
20
0
Normal (n=50)
Tumor (n=371)
TCGA samples
b)
Expression of MAPK3 in LIHC based on individual cancer stage
*
****
****
****
**
Transcript per million
80
60
40
20
0
Normal (n=50)
Stage1 (n=168)
Stage2 (n=84)
Stage3 (n=82)
Stage4 (n=6)
TCGA samples
c)
rho = 0.45
p = 6.96e-20
PRMT5 Expression Level (log2 TPM)
6
5
4
3
2
3
4
5
6
MAPK3 Expression Level (log2 TPM)

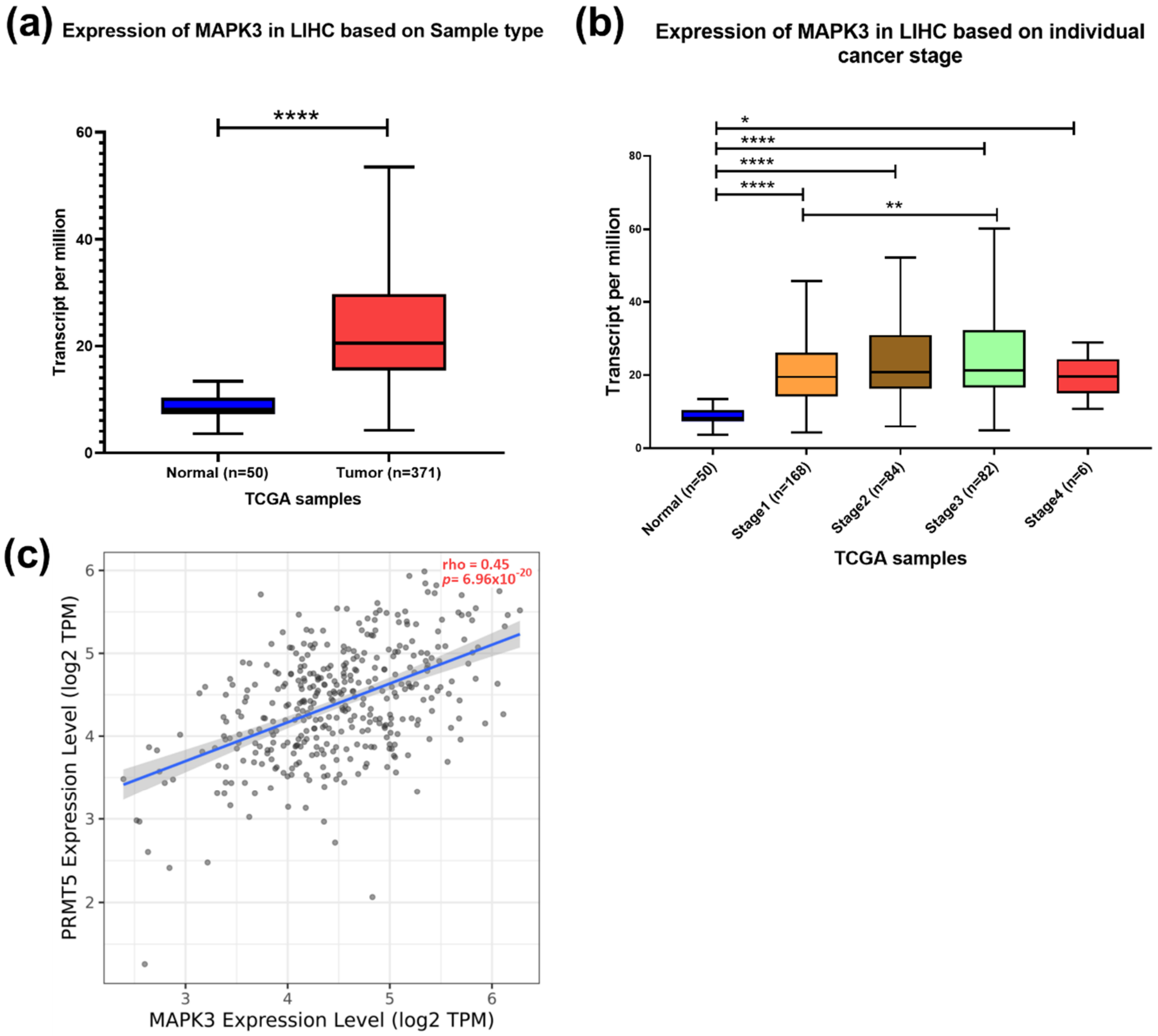

Supplementary Fig. 4: Expression and correlation of MAPK3 vs. PRMT5 in HCC. **(a)** MAPK3 expression in HCC compared to normal tissue counterpart. **(b)** MAPK3 expression in different stages of HCC compared to normal tissue counterpart. **(c)** Spearman's rank correlation coefficient of PRMT5 expression and MAPK3.

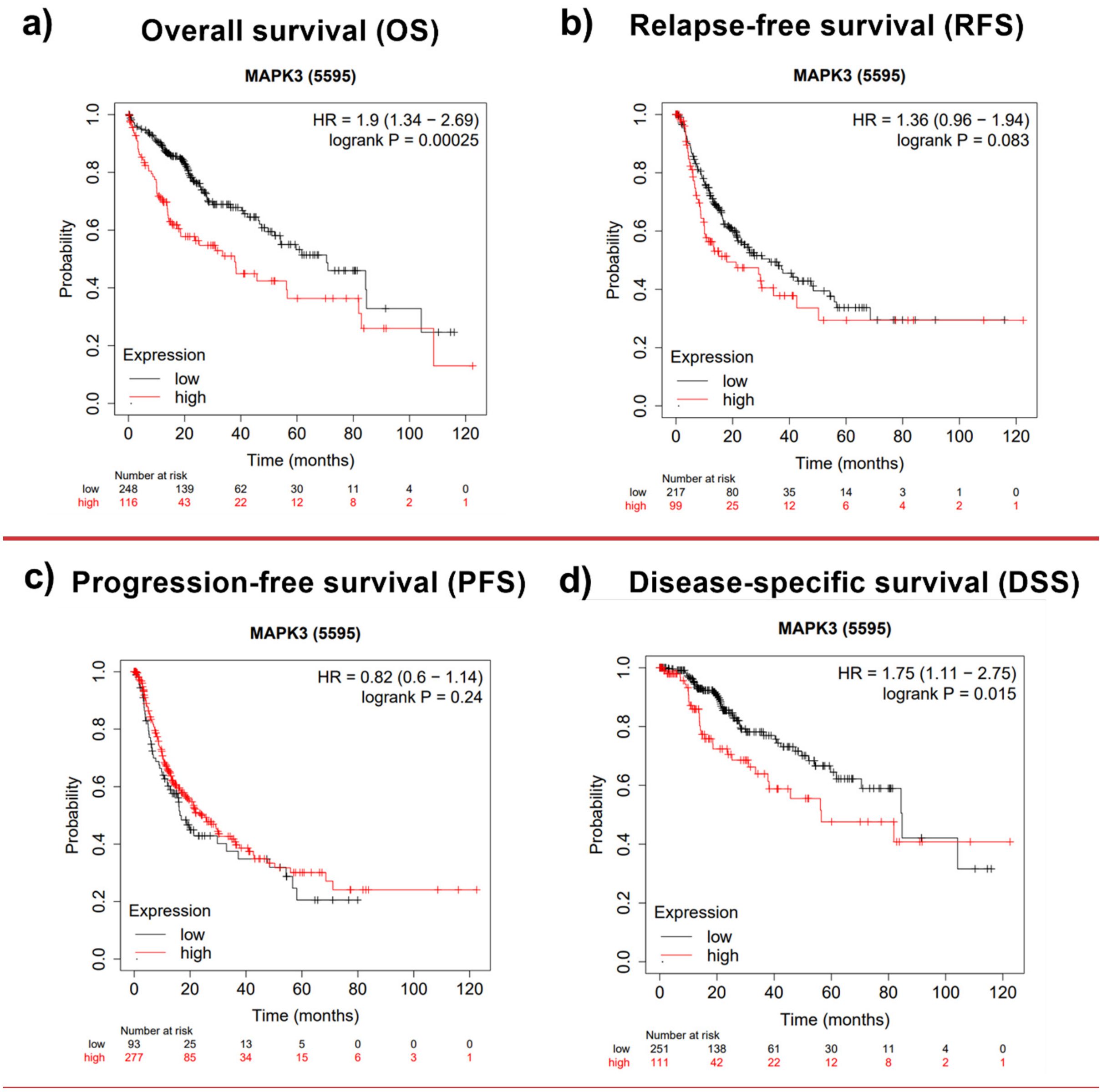
a) Overall survival (OS)
MAPK3 (5595)
HR = 1.9 (1.34 − 2.69)
logrank P = 0.00025
Expression
low
high
Probability
Time (months)
Number at risk
low 248 139 62 30 11 4 0
high 116 43 22 12 8 2 1
b) Relapse-free survival (RFS)
MAPK3 (5595)
HR = 1.36 (0.96 − 1.94)
logrank P = 0.083
Expression
low
high
Probability
Time (months)
Number at risk
low 217 80 35 14 3 1 0
high 99 25 12 6 4 2 1
c) Progression-free survival (PFS)
MAPK3 (5595)
HR = 0.82 (0.6 − 1.14)
logrank P = 0.24
Expression
low
high
Probability
Time (months)
Number at risk
low 93 25 13 5 0 0 0
high 277 85 34 15 6 3 1
d) Disease-specific survival (DSS)
MAPK3 (5595)
HR = 1.75 (1.11 − 2.75)
logrank P = 0.015
Expression
low
high
Probability
Time (months)
Number at risk
low 251 138 61 30 11 4 0
high 111 42 22 12 8 2 1

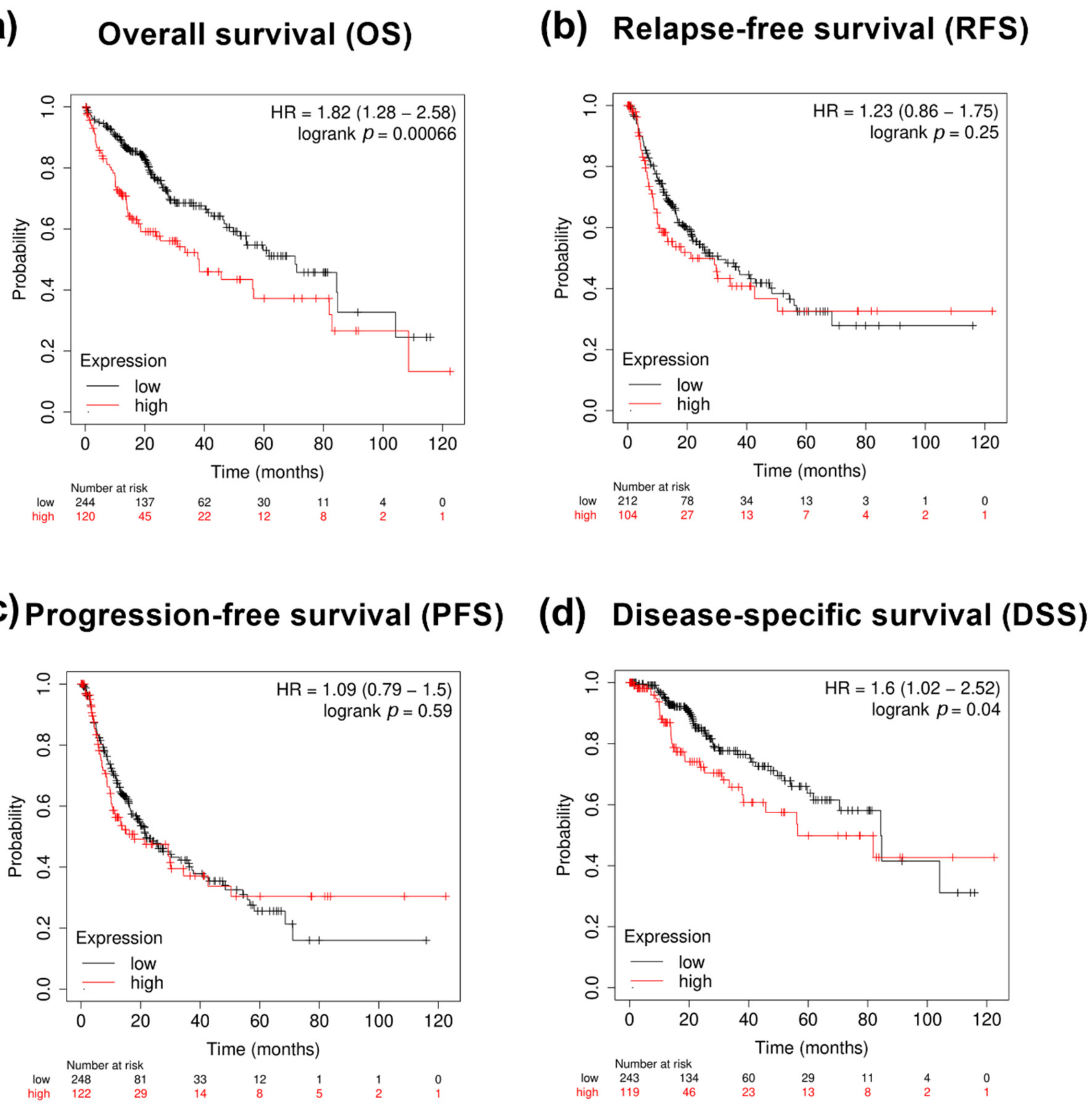

Supplementary Fig. 5: Correlational analysis of MAPK3 expression versus HCC patient survival. **(a)** MAPK3 expression versus overall patient survival (OS). **(b)** MAPK3 expression versus relapse-free survival (RFS). **(c)** MAPK3 expression versus progression-free survival (PFS). **(d)** MAPK3 expression versus disease-specific survival (DSS).
